# Development of NutVal version 5: a spreadsheet application for planning and monitoring humanitarian food assistance

**DOI:** 10.1017/S136898002610305X

**Published:** 2026-07-09

**Authors:** Andrew J. Seal

**Affiliations:** Institute for Global Health, UCL, UK

**Keywords:** Humanitarian, Food assistance, Diet planning, Micronutrients, Nutritional requirements

## Abstract

**Objective:**

To develop a software application for planning and monitoring the nutritional content and cost of humanitarian food assistance, provided by food or cash transfers.

**Design:**

Development of software.

**Setting:**

Humanitarian emergencies

**Subjects:**

Food rations or cash transfers provided to beneficiaries

**Results:**

NutVal v5 is an Excel application for the calculation of the macro- and micronutrient content of food assistance and comparison with the requirements of fourteen different population groups, including the population-averaged requirements, child and adult sub-groups, and people in detention. The criteria for nutrient inclusion and the calculation of population group requirements are presented. Market costs can be entered for the calculation of a cash-transfer equivalent value, and different food rations can be saved and compared over time.

**Conclusions:**

The NutVal application has been widely used by humanitarian organisations to plan and monitor food transfers. The enhancements in version 5 should allow for its continued use in food assistance programmes where cash and voucher transfers are increasingly common.

Globally, food insecurity and malnutrition continue to affect many populations and exert a negative impact on health, mortality and human capital^(1)^. During many humanitarian emergencies, the provision of food assistance is considered to be one of the key interventions to alleviate suffering and prevent excess mortality, and providing nutritional adequate food assistance has long been recognised as a critical public health intervention^(2–4)^. However, typical general rations have often only included a staple such as maize, legumes (beans or peas), vegetable oil and salt. Rations such as these do not provide adequate nutrients to beneficiaries and have been associated with outbreaks of micronutrient deficiencies.

The micronutrient content of general food rations is often enhanced by the inclusion of fortified vegetable oil and salt, blended foods such as corn–soya blend, and other commodities or products. The addition of specialised nutrition products, such as micronutrient powders or lipid nutrient supplements, and optimising the composition of fortified blended foods have been suggested as strategies to improve nutrient adequacy and meet international Sphere Standards for food assistance^(5–7)^. Food assistance may also include a number of other approaches for improving the nutritional intake of target populations, including cash and voucher transfers, facilitation of home gardens, income generation, social and behaviour change communication on issues such as food selection and meal preparation, and improving market access^(8)^.

Nonetheless, the design of adequate food rations remains challenging, even if supplies or commodities and products are available. In the past, food assistance rations have often been found to be deficient in micronutrients and associated with outbreaks of micronutrient deficiencies^(9–16)^, while people in detention have been another high-risk group^(17,18)^. Micronutrient and other forms of malnutrition remain prevalent in many emergency food assistance operations, and outbreaks of micronutrient deficiencies have continued to be documented in vulnerable populations^(19,20)^. In addition, staff working in food assistance programmes now face a number of other challenges as well as nutrient inadequacy.

With the move during the last decade towards the greater use of multiple, highly fortified food products in relief operations, there is also now concern that the maximum safe level of intake may be exceeded in some circumstances^(21)^. Another, relatively new concern is about the occurrence of the double burden of malnutrition in refugee and other emergency-affected populations^(22)^. This requires agencies working on nutrition and food assistance to consider concerns about the potential impact of food assistance on the risk of overweight and obesity, as well as the imperative to prevent undernutrition. Lastly, there has been a major move towards the use of cash and voucher transfers in addition to or as a replacement for food transfers. But questions remain as to how to calculate the amount of cash that should be transferred to allow for the purchase of a nutritionally adequate diet. The design and monitoring of food assistance programmes have never been easy, but it has now also become more complex as the nature of food-based emergencies and humanitarian response has evolved.

Selecting an appropriate basket of food items and balancing their quantities so as to meet macro- and micronutrient requirements requires lengthy and complicated calculations. Various software applications have therefore been developed to help staff plan and monitor the nutritional adequacy of food assistance and provide decision support for planning. These include Cost of the Diet (CoD)^(23)^, Optimus^(24)^, Optifood^(25)^, School Meal Planner (SMP) Plus^(26)^, Enhance^(27)^ and NutVal^(28)^.

The first version of NutVal, called NutVal-H, was developed by the United Nations High Commissoner for Refugees (UNHCR) during the late 1990s, to monitor the nutritional content of refugee rations and consisted of a simple calculator spreadsheet, developed in Microsoft Excel 1997. The database contained information on energy, protein, lipid and seven vitamins and minerals from 112 food items. A closed database of nutritional values was included to prevent the use of alternative nutrient composition data in the calculation of the amount of nutrients delivered by particular rations. However, in versions from 2004 onwards, a feature was added to allow users to input their own food items into the calculator. Since 2007, users have been able to download the latest versions, make suggestions for improvements and report bugs online^(28)^.

The previous version of NutVal (version 4.1) was released online in December 2015 and, up to November 2024, it had been downloaded over 10 000 times according to tracking statistics^(29)^. Users included staff of humanitarian organisations that work in food assistance, such as the World Food Programme, UNHCR and ICRC, and a range of educational institutions. The continued and widespread use of NutVal, combined with user request for enhancements, pointed to the need to provide an updated version of the software. The objective of this paper is to describe the design, development and testing of the current version of NutVal (version 5) and to lay out the calculation methods, required assumptions and some of the challenges in using the available food composition and nutritional requirement data in its construction.

## Software description

### Design principles

To ascertain what functions would be considered useful for inclusion in NutVal v5, a series of engagement activities were conducted with current or potential users of the application. These included a seminar conducted for the Cash and Voucher Assistance Working Group of the Global Nutrition Cluster in December 2023, an online questionnaire hosted at www.nutval.net, and a series of one-to-one conversations between the author and key informants experienced in humanitarian nutrition. Findings from these activities identified the need to include functions to calculate the cost of providing food assistance via cash or voucher transfers, the need to expand the list of foods included in the NutVal database, and the utility of adding a set of nutrient requirements for people in detention. In addition, users wished to be able to adjust the nutrient requirement target figures according to the degree of dependency on food assistance. These features were subsequently included in the revision described below.

During the development of version 5, it was decided to retain the user-friendly and familiar graphical user interface (GUI) of Excel, an application used almost ubiquitously by humanitarian aid organisations. These considerations were particularly important given the international target audience and varying levels of computer literacy expected in potential users. The main changes in the application, starting with NutVal 2006, are documented for each version on the ‘What’s New in NutVal XX’ help page within the application.

### Use of Excel and Visual Basic for Applications

The application was built in Microsoft Excel using Visual Basic for Applications (VBA) code to automate calculations and manipulate Excel objects^(30)^. The standard Excel GUI was modified to add application-specific functions and simplify use, without losing the familiar look of Excel. Excel form controls were used extensively to simplify navigation between different worksheets and for running macros that carry out calculations or manipulate data. To simplify coding and make updates easier, extensive use was made of named ranges. A simple food item database was included as a hidden worksheet, and a data storage and retrieval function was developed to facilitate the saving and recall of different food rations and costs. Comparative analysis of different food assistance rations was enabled by inclusion of automated pivot tables and pivot charts. Worksheets were password-protected, cells were locked and the names or named ranges were hidden to reduce the risk of user confusion or inadvertent changes to functionality.

### User interface and functions

**Figure 1.**
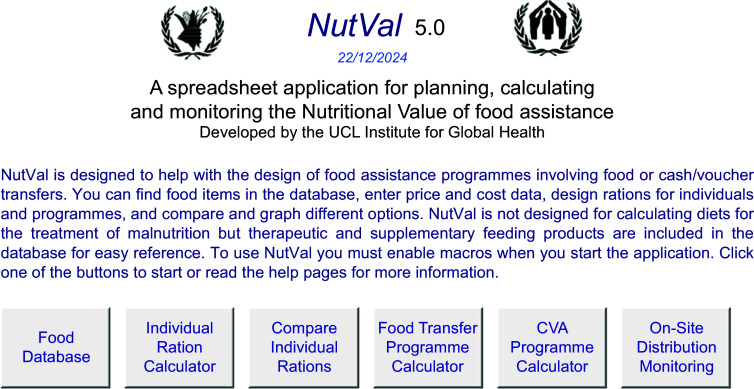
The application home screen.

Figure 1 shows the ‘home’ screen from which users can navigate to other worksheets containing a food list, a calculator for individual requirements, a ration comparison table and food and cash-transfer calculators to calculate the amounts and costs involved in scaling up an individual ration to that required for beneficiary populations over different time periods.

**Figure 2.**
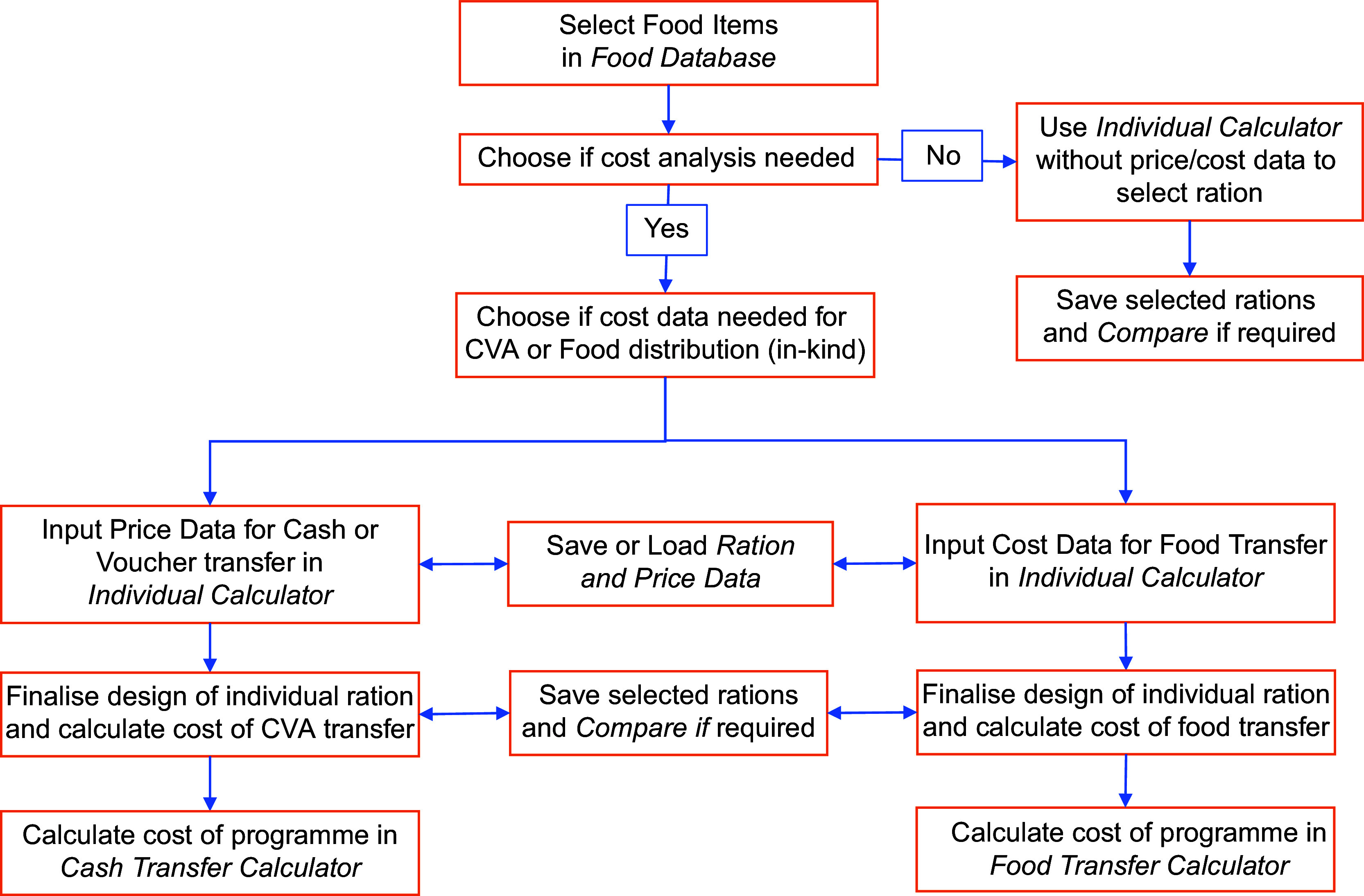
Figure 2 long description. Workflow schematic and available functions in NutVal 51.

Figure 2 shows the design of the application workflow, with the different NutVal functions (worksheets) shown in italics and decision points for users in blue. After the initial selection of food items for inclusion in the ration, the user decides if cost analysis is required. If so, the cost and/or prices for the selected food items are entered or loaded (if previously saved), and the ration is adjusted to ensure it meets the requirements of the target population at an acceptable cost. The individual ration can then be saved and compared to other options or previously used rations if required. Finally, the selected individual ration can be viewed in one of the programme calculators, where the quantities and costs are scaled up according to the size of the beneficiary population and the planned duration of the programme.

### Selection of nutrients for inclusion in database

A wide range of human nutrients were reviewed for possible inclusion in the NutVal database. The final selection of twenty-one nutrients and energy was made in accordance with those selected for inclusion in the 2011 version of the Sphere Project humanitarian standards^(5)^. This selection was based on a review of nutrient requirement references and norms for emergency-affected populations, supplemented by an online consultation process as previously described^(7)^. They included all micronutrients associated with previously described outbreaks of micronutrient deficiencies in emergency-affected populations, together with other minerals and vitamins with well-described physiological functions. To retain the simplicity necessary for use by a wide range of humanitarian staff, only the basic categories of macronutrients were included. The limitations of this approach for defining long-term health risks, such as CVD, were considered, but, on balance, it was decided to not include sub-categories of lipids or measures of protein quality.

### Selection of population groups

Food assistance programmes may be designed to meet the nutritional needs, in whole or in part, of the general population or specified sub-population groups. General rations (also called general food distributions) are designed for the whole population. As detailed and accurate information on the demographic composition of the population is usually not available, the requirements for the whole population are based on a predefined population composition and assumptions about the level of physical activity and the ambient temperature^(31,32)^.

The selection of population sub-groups for inclusion was based, first, on consideration of how different a sub-group’s nutrient requirements were in comparison to the general population and, second, on which population groups are usually targeted by humanitarian nutrition programmes. Typically, the age groups targeted in child-focused nutrition programmes include 6–59 months or 6–23 months. Pregnant and lactating women are often included as target for blanket supplementary feeding programmes. Other types of programmes are aimed at adult detainees, such as prisoners of war, or other vulnerable groups.

### Calculation and compilation of population group nutrient requirements

The energy requirements to be used for the initial planning of a ration for an emergency-affected general population were defined by UN agencies and published in 2002^(31)^. The energy requirements for population sub-groups were calculated based on the requirements given in: Human Energy Requirements, Report of a Joint FAO/WHO/UNU Expert Consultation, Rome, 17–24 October 2001^(33)^. Where the population sub-groups in the report differed from those used in NutVal, weighted averages were calculated. An excessive energy content of a selected ration was defined as being greater than 115 % of the calculated requirement.

The requirements for protein and fat are taken from the 2002 publication, ‘Food and Nutrition Needs in Emergencies’^(31)^. This multi-agency UN publication specifies the need to supply a minimum of 10 %, and a maximum of 15 %, of dietary energy intake as protein, and to supply at least 30 % of dietary energy as fat for children of 6–23 months, 20 % for pregnant and lactating women, and 17 % for other groups. Using these percentages and the energy requirements calculated for each beneficiary group, the corresponding requirements for protein and fat were calculated.

Micronutrient requirements for the Whole Population planning figure were calculated based on the requirements detailed in: Vitamin and Mineral Requirements in Human Nutrition, Second edition, WHO/FAO (2004), except for copper, which was taken from Trace Elements in Human Nutrition and Health, WHO, 1996^(34,35)^. The figures are identical to those used in the 2011 and 2018 editions of The Sphere Project, Humanitarian Charter and Minimum Standards in Humanitarian Response. The methods used for calculation have been previously published^(7)^.

When deriving micronutrient requirements for population sub-groups, it was observed that neither the FAO nor the WHO age groups were suited for use in humanitarian nutrition programme planning. To allow for calculation of requirements for the age groups used in NutVal, weighted averages of the appropriate age group requirements were used. The calculations assumed equal proportions of males and females.

The application includes the target requirement for fourteen population groups. These include requirements for: (a) a planning ration designed for use when deciding on general rations for the whole population, (b) age-defined population sub-groups, (c) women who are pregnant or lactating and (d) the special group ‘people in detention’, which is designed to be used by humanitarian organisations or prison authorities when planning rations for prisoners or detainees. Prisoners of war and other categories of detainees are a special focus of organisations such as the International Committee of the Red Cross (ICRC). Following consultations and discussions with nutrition experts at ICRC, it was decided to set the nutritional requirements for this group to 2,400 kcal, with a proportionate inclusion of macro and micronutrient requirements from the general ration.

Table 1 shows the minimum levels of energy, protein, and fat required by the different beneficiary groups. The calculated protein and fat requirements are provided in terms of g/person/d and as a minimum proportion of the provided energy. The calculated requirements and upper tolerable limits (UL) for minerals and vitamins are given in Tables 2 and 3.

**Table 1.** Energy and macronutrient requirements for population groups included in NutVal Table 1 long description.

Population group	Energy (kcal/person/d)	Minimum protein (g/person/d)^1^	Acceptable range of energy from protein (%)	Minimum fat (g/person/d)	Acceptable range of energy from fat (%)
Whole population	2100	52.5	10–15	39.7	17–30
6–23 months	834	20.8	10–15	27.8	30–40
24–59 months	1198	29.9	10–15	22.6	30–40
6–59 months	1076	26.9	10–15	20.3	30–40
5–9 years	1640	41.0	10–15	31.0	17–30
10–14 years	2406	60.1	10–15	45.4	17–30
15–17 years	2901	72.5	10–15	54.8	17–30
18–59 years	2511	62.8	10–15	47.4	17–30
18–59-year-old males	2793	69.8	10–15	52.8	17–30
18–59-year-old females	2229	55.7	10–15	42.1	17–30
≥60 years	2175	54.4	10–15	41.1	17–30
Pregnant women	2718	67.9	10–15	60.4	20–30
Lactating women	2900	72.5	10–15	64.4	20–30
People in detention	2400	60.0	10–15	45.7	17–30

**Table 2.** Mineral requirements and upper tolerable intake levels calculated for population and sub-groups^*^

Population group		Ca (mg)	Cu (mg)	I (μg)	Fe (mg)	Mg^†^ (mg)	Se (μg)	Zn (mg)
Whole population	RNI	989	1.1	138	32.0	201	27.6	12.4
	UL	2000	10.0	600	45.0	–	400	34
6–23 months	RNI	472	0.6	90	13.9	58	15.1	8.3
	UL	1500	1.5	200	40.0	–	60	5.0
24–59 months	RNI	564	0.6	90	12.6	70	20.2	9.1
	UL	1500	1.5	200	40.0	–	90	7.0
6–59 months	RNI	526	0.6	88	12.9	65	18.3	8.7
	UL	1500	1.5	200	40.0	–	60	5.0
5–9 years	RNI	660	0.7	108	16.0	90	21.4	10.6
	UL	2500	3.0	250	40.0	–	150	12.0
10–14 years	RNI	1300	1.0	140	40.1	225	29.0	15.8
	UL	3000	6.0	450	40.0	–	280	23.0
15–17 years	RNI	1300	1.0	140	40.1	225	29.0	15.8
	UL	3000	10.0	500	45.0	–	400	34.0
18–59 years	RNI	1039	1.2	150	39.1	240	30.0	12.0
	UL	2500	10.0	600	45.0	–	400	34.0
18–59-year-old males	RNI	1007	1.3	150	27.2	259	34.0	14.1
	UL	2000	10.0	600	45.0	–	400	34.0
18–59-year-old females	RNI	1071	1.1	150	51.0	220	26.0	9.9
	UL	2000	10.0	600	45.0	–	400	34.0
≥60 years	RNI	1300	1.3	150	25.0	207	29.0	11.9
	UL	2000	10.0	600	45.0	–	400	40.0
Pregnant women	RNI	1067	1.2	200	30.0	220	28.0	15.0
	UL	2500	10.0	600	−^‡^	–	400	40.0
Lactating women	RNI	1000	1.3	200	30.0	270	38.5	21.1
	UL	2500	10.0	600	−^‡^	–	400	40.0
People in detention	RNI	1130	1.3	158	36.6	230	31.5	14.2
	UL	2000	10.0	600	45.0	–	400	34.0

UL, upper tolerable limits.

* All requirements are given per person per day.

† UL were based on intakes from supplements rather than foods, so they were not included.

‡ Values for UL were not available for pregnant or lactating women.

**Table 3.** Vitamin requirements and upper tolerable intake levels calculated for population sub-groups^*^ Table 3 long description.

		A (μg)	B_1_^†^ (mg)	B_2_^†^ (mg)	B_3_ (mg)	B_5_^†^ (mg)	B_6_ (mg)	B_9_^‡^ (μg DFE)	B_12_^†^ (μg)	C (mg)	D (μg)	E (mg)	K^†^ (mg)
Whole population	RNI	550	1·10	1·10	13·8	4·6	1·2	363	2·2	41·6	6·1	8·0	48·2
	UL	1171	–	–	263	–	8·8	–	–	701	72	140	–
6–23 months	RNI	400	0·44	0·47	5·4	1·9	0·4	131	0·8	30·0	5·0	4·4	13·6
	UL	600	–	–	150	–	5·0	–	–	400	38	100	–
24–59 months	RNI	432	0·56	0·56	7·3	2·6	0·6	182	1·1	30·0	5·0	5·0	18·2
	UL	800	–	–	150	–	5·0	–	–	400	38	100	–
6–59 months	RNI	415	0·52	0·53	6·6	2·4	0·5	163	1·0	29·4	4·9	4·7	16·5
	UL	600	–	–	150	–	5·0	–	–	400	38	100	–
5–9 years	RNI	480	0·78	0·78	10·4	3·6	0·8	260	1·6	33·0	5·0	6·2	23·0
	UL	1100	–	–	220	–	7·0	–	–	650	75	120	–
10–14 years	RNI	600	1·15	1·15	16·0	5·0	1·3	400	2·4	40·0	5·0	8·8	45·0
	UL	1500	–	–	350	–	10	–	–	1200	100	160	–
15–17 years	RNI	600	1·15	1·15	16·0	5·0	1·3	400	2·4	40·0	5·0	8·8	45·0
	UL	2600	–	–	700	–	20	–	–	1800	100	260	–
18–59 years	RNI	551	1·15	1·20	15·0	5·0	5·0	400	2·4	44·9	6·0	8·8	59·6
	UL	3000	–	–	900	–	20	–	–	2000	100	300	–
18–59-year-old males	RNI	600	1·20	1·30	16·0	5·0	5·0	400	2·4	44·9	7·0	10·0	64·5
	UL	3000	–	–	900	–	20	–	–	2000	100	300	–
18–59-year-old females	RNI	502	1·10	1·10	14·0	5·0	5·0	400	2·4	44·9	6·0	7·5	54·8
	UL	3000	–	–	900	–	20	–	–	2000	100	300	–
≥60 years	RNI	600	1·15	1·20	15·0	5·0	1·6	400	2·4	45·0	15·0	8·8	60·0
	UL	3000	–	–	900	–	25	–	–	2000	100	300	–
Pregnant women	RNI	800	1·40	1·40	18·0	6·0	1·9	600	2·6	55·0	5·0	7·5	55·0
	UL	3000	–	–	−^§^	–	25	–	–	2000	100	300	–
Lactating women	RNI	850	1·50	1·60	17·0	7·0	2·0	500	2·8	70·0	5·0	7·5	55·0
	UL	3000	–	–	−^§^	–	25	–	–	2000	100	300	–
People in detention	RNI	629	1·26	1·26	15·8	5·3	1	415	3	47·5	7·0	9·1	55·1
	UL	1171	–	–	263	–	8·8	–	–	701	72	140	–

UL, upper tolerable limits.

* All requirements are given per person per day.

† Values for UL were not available.

‡ No UL values were available for dietary folate equivalents derived from food sources.

§ Values for niacin UL were not available for pregnant or lactating women.

### Selection and compilation of upper tolerable limits

The UL for the nineteen nutrients included in NutVal were chosen from four key references: WHO/FAO (2005), the European Food Standards Authority (EFSA) (2006), reports from the US Institute of Medicine (IOM) (various dates, 1997–2011) and the UK Expert Group on Vitamins and Minerals (2003)^(35–38)^. FAO/WHO was the preferred reference due to its global scope, the normative mandate of WHO and, therefore, its applicability to users of NutVal, who work across a wide range of countries. However, age group-specific UL were not specified by FAO/WHO for many nutrients. The EFSA was used as the second preference reference, given its regional nature, followed, if necessary, by one of two sources with a national focus, the IOM, which sets references for the USA and Canada, or the UK Expert Group on Vitamins and Minerals. Where possible, one reference was used per nutrient for setting upper limits across all age stages and life groups. The date of publication was also taken into account in the selection process. A systematic approach was developed for selection of UL for different nutrients and is illustrated in Supplementary Figure 1.

### Compilation of food and nutrient database

The database of food items was expanded in version 5 by the inclusion of eighty-two additional foods (total 270). The selection of items to include was done in consultation with ICRC nutrition staff and other key informants. Foods were prioritised for inclusion if they were often included in food assistance programmes or they formed a core part of the diet in emergency-affected populations. In addition to energy, protein and fat, data on seven minerals and twelve vitamins were included in NutVal v5. Some missing nutrient data were found for most food items, with certain nutrients being most likely to have missing values. For example, values for vitamins B_1_, B_2_ and niacin content were available for 99 % of all food items, while the value for iodine was only available for 15 % of items. Missing data were represented in the NutVal food database by a dash.

Nutrient values for most food were taken from the US Department of Agriculture food composition databases, available at FoodData Central^(39)^. Additional items were included from the FAO/INFOODS Food Composition Table for Western Africa^(40)^. The composition of camel milk was taken from Medhammar *et al.* (2012) and Sadler *et al.* (2009)^(41,42)^. Generic composition data for canned cheese, meat and dried fish were included from ‘Food and Nutrition Needs in Emergencies’ (2002)^(31)^. Specifications were provided by WFP, United States Agency for International Development, or UNICEF for a number of different nutritional products. WFP specifications for fortified foods were derived from the results of shelf-life studies conducted by WFP under varying heat and humidity conditions^(43)^. Specifications for micronutrient powders for children aged 6–59 months and schoolchildren were set by WFP in 2023^(44,45)^.

### Calculation of cash-transfer values for food assistance programmes

When planning cash or voucher transfer programmes, retail price data can be used to calculate the value needed for an individual or household to purchase a specified diet. NutVal is designed so that data on average market prices can be entered and saved for each food item. Users are also recommended to enter an estimate for the expected inflation or deflation rate over the course of the planned programme, and, if this is done, the software calculates an adjusted value for the required cash transfer, assuming that prices increase or decrease as a linear function over the time period of the programme.

### Calculation of costs for direct (in kind) food transfer programmes

To calculate costs for food transfers, NutVal uses values for the different categories of costs that are entered by the user. The categories included are those used by WFP, that is, procurement cost, shipping cost, and the cost for landside transport, storage and handling.

### Beta testing and release

Testing of version 5 was undertaken by staff from the ICRC over a period of several weeks in autumn 2024. Two webinars were held with invited staff: the first to introduce the new test version and the second, one after several weeks of trial use, to garner feedback from users. Following this piloting, further revisions were made. The final version was released online on 24 December 2024 at www.nutval.net. Users are encouraged to provide feedback, request changes and report any bugs via the website. A user group had been previously established to facilitate users being kept aware of updates and other relevant developments.

## Discussion

The development of NutVal version 5 has provided additional functionality for designing and monitoring food and cash-transfer programmes and is expected to allow for improved analysis and decision-making. Future work will aim to investigate whether the added functionality results in improvements in nutritional composition and adequacy and more cost-effective use of resources.

During the design of the application, data had to be compiled on nutritional requirements, food composition and upper tolerable intakes. In addition to compiling these parameters, it was necessary to develop calculation methods and associated assumptions in order to generate population-level requirements. This process revealed a number of challenges with the currently available nutritional data and approaches to developing normative guidance on requirements.

One challenge that was encountered was that the age and sex population groups used by nutrient requirement setting committees did not usually match up with the age groups commonly used in planning and implementing nutrition programmes. Age group-specific requirements therefore had to be generated by using weighted averages and interpolation. While this was usually straightforward, in some cases a judgement had to be made as to the best way to derive the requirements.

As well as encouraging the design of food assistance that provides minimum levels of essential nutrients, it was also considered important that NutVal users were made aware of when excessive intakes of micronutrients might occur. A process was therefore undertaken to compile the available UL published by different authoritative bodies and select the most appropriate figures to include in the calculator. It was found that UL were not available for all nutrients and population groups, so in these circumstances, they had to be omitted. In some cases, no adverse effects had been noted by the UL review committees, for example, thiamine, riboflavin and pantothenic acid, so UL were not published, and in other cases, vitamins B_12_ and K, there was not adequate data for them to be derived.

In the case of Mg and folate, UL were designed to only apply to supplementary intakes rather than to the content of the unsupplemented food, so they were not applicable for use in ration calculation and not included in NutVal. No body had set UL for vitamin B_3_ (niacin) during pregnancy or lactation, so no value could be included. Finally, in the case of iron, while UL were available for women undergoing pregnancy or lactation, these were lower than recommended prophylactic doses for iron deficiency anaemia. To prevent potential confusion about the safety of using iron-fortified foods in feeding programmes for pregnant and lactating women, it was decided to not include the iron UL for these two groups.

The revision and publication of UL is an ongoing process, and new UL are expected to be published by the European Food Safety Authority in the near future and will be considered for inclusion in future NutVal updates^(46)^. Ideally, the processes by which RNI and UL are derived in the future will be more closely linked and performed by the same expert committees. This would allow for a more holistic consideration of the risks and benefits of different levels of nutrient consumption and might help prevent situations where the RNI and UL may end up being set so close to each other that their application in practical diet planning tools and programmes becomes problematic.

Use of the NutVal application illustrates the problem of achieving adequate nutrient intakes with commonly available food assistance commodities and products. The problem of general ration design has long been acknowledged^(8)^. For example, designing rations with adequate Ca content is particularly difficult to achieve in the absence of dairy products, even when including the fortified foods that are currently available. When designing food aid products and formulating fortificant mixes in the future, it would be helpful if ration planning tools, such as NutVal, were used to assess the adequacy and safety of typical food assistance rations that can be designed with the proposed products.

The expansion of the food item database involved including data from a number of food composition databases. It was found that the United States Department of Agriculture and the FAO/INFOODS Food Composition Table for Western Africa databases were the most useful sources of data for the identified items^(39,40)^. However, while the NutVal database has been significantly expanded, there will still inevitably be situations in which users wish to include items that are not in the database. The ability for the user to enter other food items into the calculation sheet was introduced in a previous NutVal update, and this was maintained in version 5, with up to twenty additional rows allowed for user-specified items.

It is appreciated that users may be working in situations where national or local dietary guidelines may differ from the requirements that are specified by WHO/FAO and included in NutVal. It can be argued that it would be useful for users to be able to adjust the application’s requirement figures in these situations, as this would allow for the use of NutVal in a wider variety of nutritional planning contexts. However, the counterargument is that requirements for emergency-affected populations should be based on international guidance, such as that provided by WHO and the Sphere Project, and fixed to ensure operational harmonisation and counter possible manipulation by parties to a conflict or other stakeholders. The latter position was adopted during the development of version 5.

While NutVal is designed to help users design adequate food assistance, it is appreciated that resources may not always be available to allow for requirements to be met, requiring difficult choices to be made. Depending on the available food items, it may be particularly expensive to achieve adequacy for particular nutrients, for example, Ca or vitamin B_12_. In the absence of animal products, fortified foods may be required, and these may be expensive and/or limited in quantity. Therefore, users may need to make a trade-off between the cost and the nutritional content of the planned food assistance. While version 5 does not provide specific guidance in these situations, it is intended that a future update will build in decision support for those contexts, helping the user to prioritise the optimisation of nutrients that will reduce the risk of deficiencies with the highest public health impact. Nutrient prioritisation will also aim to take into account the selected staple food and the associated risks of deficiency disease.

As with many diet planning tools, assumptions are made that the intra-household distribution of food takes places in a way that optimises nutrient intakes for all household members. In a similar way, food preparation methods are also assumed to lead to minimal nutrient loss and that any bartering or sale of received food does not result in a reduction in the overall availability of nutrients. In reality, none of these assumptions may hold true, and post-distribution monitoring is required to understand food utilisation in each operational context. Adjustments to the ration plan may be required based on these results, and this should also take into account the contribution of any supplementary feeding programmes and other sources of food assistance.

NutVal version 5 can be used to calculate the value of a cash transfer that is required to meet a household’s nutritional needs. Additional assumptions and monitoring are required in this case. It is necessary to set the expected inflation rate and monitor the actual rate for different food items, understand the proportion of unconditional cash transfers that are spent on food and track the seasonal availability of foods in the market.

While there has been a marked drop-off in the reporting of major micronutrient deficiency outbreaks in recent years, it is difficult to assess whether this is only due to improved nutritional adequacy or whether less attention and data collection are also important factors. In the current nutritional emergencies in Gaza and Sudan, there have been protracted sieges and deprivation. While little data on micronutrient malnutrition are available for these contexts, the reported outbreak of neuropathy in Gaza is suggestive of severe B vitamin deficiencies^(47)^. In contexts such as these, a renewed analytical focus on micronutrient adequacy using tools such as NutVal, as well as starvation, is urgently required.

The severe cuts in humanitarian funding over the last year or so have resulted in a deterioration in the availability of food assistance. While there is mounting concern over availability, the nutritional adequacy of food assistance should also be monitored. This will be important to document the impact of the cuts, monitor changes in nutritional adequacy and serve as an early warning approach to try and ensure that we do not return to an era of large-scale micronutrient deficiency outbreaks in crisis-affected populations. In this time of particular resource scarcity, achieving nutritional adequacy at the lowest possible cost is essential as efforts to assist the most vulnerable populations continue.

## Supporting information

10.1017/S136898002610305X.sm001 Seal supplementary material Seal supplementary material

## Supplementary material

For supplementary material accompanying this paper visit https://doi.org/10.1017/S136898002610305X

## Long description

The flowchart illustrates the process of selecting food items and analyzing costs for food assistance programs. Panel A: The process begins with selecting food items in the Food Database. Next, a decision is made whether cost analysis is needed. If no, the user proceeds to use the Individual Calculator without price/cost data to select rations. If yes, another decision is made whether cost data is needed for CVA or Food distribution (in-kind). If yes, the user inputs price data for Cash or Voucher transfer in the Individual Calculator or inputs cost data for Food Transfer in the Individual Calculator. The user then saves or loads ration and price data. The design of the individual ration is finalized, and the cost of CVA transfer or food transfer is calculated. The selected rations are saved and compared if required. Finally, the cost of the program is calculated in the Cash Transfer Calculator or Food Transfer Calculator.

Navigate back to .

## Table 1. Long description

A table comparing energy and macronutrient requirements for different population groups. The table has 13 rows and 6 columns. The columns are labeled as Population group, Energy (kcal/person/d), Minimum protein (g/person/d), Acceptable range of energy from protein (%), Minimum fat (g/person/d), and Acceptable range of energy from fat (%). The rows list different population groups and their corresponding energy and macronutrient requirements. Row 1: Whole population, 2100, 52.5, 10-15 percent, 39.7, 17-30 percent. Row 2: 6-23 months, 834, 20.8, 10-15 percent, 27.8, 30-40 percent. Row 3: 24-59 months, 1198, 29.9, 10-15 percent, 22.6, 30-40 percent. Row 4: 6-59 months, 1076, 26.9, 10-15 percent, 20.3, 30-40 percent. Row 5: 5-9 years, 1640, 41.0, 10-15 percent, 31.0, 17-30 percent. Row 6: 10-14 years, 2406, 60.1, 10-15 percent, 45.4, 17-30 percent. Row 7: 15-17 years, 2901, 72.5, 10-15 percent, 54.8, 17-30 percent. Row 8: 18-59 years, 2511, 62.8, 10-15 percent, 47.4, 17-30 percent. Row 9: 18-59-year-old males, 2793, 69.8, 10-15 percent, 52.8, 17-30 percent. Row 10: 18-59-year-old females, 2229, 55.7, 10-15 percent, 42.1, 17-30 percent. Row 11: ≥ 60 years, 2175, 54.4, 10-15 percent, 41.1, 17-30 percent. Row 12: Pregnant women, 2718, 67.9, 10-15 percent, 60.4, 20-30 percent. Row 13: Lactating women, 2900, 72.5, 10-15 percent, 64.4, 20-30 percent. Row 14: People in detention, 2400, 60.0, 10-15 percent, 45.7, 17-30 percent.

Navigate back to Table 1..

## Table 3. Long description

The table compares vitamin requirements and upper tolerable intake levels for various population sub-groups. It has 14 rows and 12 columns. The columns are labeled as follows: A (µg), B1 (mg), B2 (mg), B3 (mg), B5 (mg), B6 (mg), B9 (µg DFE), B12 (µg), C (mg), D (µg), E (mg), K (mg). The rows are labeled as follows: Whole population, 6-23 months, 24-59 months, 6-59 months, 5-9 years, 10-14 years, 15-17 years, 18-59 years, 18-59-year-old males, 18-59-year-old females, ≥ 60 years, Pregnant women, Lactating women, People in detention. Each row provides specific values for the vitamin requirements and upper tolerable intake levels for the respective population sub-group.

Navigate back to Table 3..
