# Supplementary material for: Development of NutVal version 5: a spreadsheet application for planning and monitoring humanitarian food assistance
Source: Public Health Nutr. 2026 Jul 9;29(1):e166. doi: 10.1017/S136898002610305X (PMC13468943; doi:10.1017/S136898002610305X)
Supplement: Supplementary file 1 [file S136898002610305Xsup001.docx]

**Web** **Figure 1: Algorithm Used for Selecting Tolerable Upper Intake Levels (UL) for Vitamins and Minerals**

Does WHO/FAO provide any age group/life stage specific ULs?

Yes

No

Does EFSA provide any age group/life stage specific ULs?

Yes

No

Does IOM provide any age group/life stage specific ULs?

Yes

No

Does FAO/WHO provide ULs for all* age groups/life stages?

Yes

No

Next

Does IOM provide ULs for all* age groups/life stages?

Yes

No

Does EFSA provide ULs for all* age groups/life stages?

Yes

No

Enter in to

NutVal database

Is it the most up to date of the references with all* ULs?

Yes

No

Is it the most up to date of the references with all* ULs?**

Yes

No

No

Is it the most up to date of the references with all* ULs?

Is it the most up to date of the references with all* ULs?

Yes

Yes

Is it the most up to date of the references for that specific age/group/life stage?

Yes

Yes

No

Is it the most up to date of the references for that specific age/group/life stage?

Enter in to

NutVal database

Next

Enter in to

NutVal database

Next

Next

Enter in to

NutVal database

No

Enter in to

NutVal database

Leave remaining blank

Is it the most up to date of the references for that specific age/group/life stage?

No

Yes

Enter in to

NutVal database

Enter in to

NutVal database

Enter in to

NutVal database

Does UK provide ULs for all* age groups/life stages?

Yes

No

No

Does UK provide any age group/life stage specific ULs?

Yes

No

*With the exception of infants 6-12 months, as only one reference setting body, IOM, routinely made recommendations for this age group where others did not for Vitamin A, calcium, and zinc. No bodies made UL recommendations for infants under 12 months for vitamins E, B3, B6, folate, vitamin C, copper, iodine, and magnesium. We therefore used the UL for children 1-3 years for the age groups that include infants under 12 months.

** Exceptions to rule:

1. Selenium: IOM ULs were chosen over EFSA ULs because the EFSA ULs are very close to the FAO/WHO RNIs. The IOM reference better recognises the existence of seleniferous areas where long term intakes may exceed ULs but with no observed toxic effects.

2. Zinc: IOM ULs chosen over EFSA ULs despite EFSA being the more up to date, full set, of ULs because the EFSA ULs are very close to the FAO/WHO RNIs.
